# Design of Drug Sales Forecasting Model Using Particle Swarm Optimization Neural Networks Model

**DOI:** 10.1155/2022/6836524

**Published:** 2022-07-04

**Authors:** Chenggong Yu

**Affiliations:** Zhejiang Pharmaceutical University, Ningbo 315000, China

## Abstract

The establishment of enterprise target inventory is directly related to the forecast of drug sales volume. Accurate sales forecasting can help businesses not only set accurate target inventory but also avoid inventory backlogs and shortages. In this paper, NN technology is used to forecast sales and is optimized using the PSO algorithm, resulting in the creation of a drug sale forecast model. The model optimizes the weights and thresholds of NN using the improved PSO optimization algorithm and predicts the periodic components based on time correlation characteristics, effectively describing the trend growth and seasonal fluctuations of sales forecast data. Furthermore, the model in this paper has been creatively improved according to the needs of practical application, which has improved the shortcomings of traditional NN, such as long training time, slow convergence speed, and ease to fall into local minima, to improve performance and quality, and has received positive results in application. The experimental results show that this model has a prediction accuracy of 96.14 percent, which is 12.78 percent higher than the traditional BP model. The optimized model can be used to forecast drug sales in a practical and feasible way.

## 1. Introduction

With the intensification of market competition, users of information systems are no longer satisfied with the fact that computers are only used for daily affairs, and they urgently need information that supports the decision-making process [1]. At the same time, in today's market, the market environment that enterprises face is so complicated and changeable as understanding the course, carrying out work actively and effectively, and making decisions are problems that an enterprise must solve [2]. The sales link, which connects suppliers, sellers, and consumers, is an important link in enterprise planning. The most important part of the sales process is sales forecasting, and most commodity circulation industries' supply chains have inventory and sales problems due to a lack of appropriate sales forecasting methods. The sales forecast is modeled using a set of highly complicated nonlinear systems that are manually defined [3]. One of the most important aspects of a company's sales plan, as we all know, is sales forecasting. It is inseparable from the planning of the future development direction, regardless of how large the company is or how complicated the sales staff is. However, because the future sales situation will inevitably and to a large extent influence the development direction, the sales forecast is almost an important pending event for enterprise managers and decision-makers related to the survival of enterprises. At present, many enterprises are still using traditional sales forecasting methods. Although the traditional sales forecasting method is widely used and the effect is good, it is difficult to deal with the complicated nonlinear relationship and various complicated influencing factors. Therefore, we need a new method [4]. Based on this, this paper will forecast and analyze the historical sales data of the sales department of the pharmaceutical group through PSONN (particle swarm optimization neural network) and provide some references for decision-makers in purchasing, selling, and storing drugs.

With the development of computer science, more and more researchers began to explore the use of artificial intelligence forecasting methods for medium and long-term load forecasting. At present, the most commonly used forecasting methods mainly include time series analysis, linear regression, nonlinear regression, grey forecasting, input-output forecasting, Markov forecasting, and ANN (Artificial neural network) [5–7] forecasting. Among several commonly used forecasting methods, the ANN forecasting method has attracted much attention in recent years. BPNN (backpropagation neural network) is a kind of commonly used ANN, which is used to train multilayer forward NN (neural network). BPNN contains the best part of NN theory, with a simple structure and strong plasticity. It adopts a Sigmoid type transfer function, and its learning mode is a multilayer feedforward network with minimum mean square error. There have been a lot of research studies on NN prediction [8, 9], but simply using NN to predict can no longer meet the real-time needs, and in the increasingly complex environment, more and more methods are needed to improve NN research. In this paper, the learning theory of PSOBPNN (particle swarm optimization backpropagation neural network) is used to establish an algorithm to predict the drug sales in the next year, and an optimization method is proposed for drug production, thus forming an effective drug production decision model. The innovations of this paper are as follows:

  ① This paper analyzes the difficulties in forecasting the sales volume of multivariety drugs in enterprises, the difficulties in the application of existing forecasting methods, and the advantages of the model in dealing with the problems and difficulties. To solve these problems, a BPNN learning algorithm is put forward. On this basis, PSOBPNN is used to forecast the sales data.
  ② In this paper, the PSO optimization algorithm is used to globally search the weights and thresholds of BPNN, and the optimized BPNN is used to establish a prediction model to predict the drug sales index. This model improves the shortcomings of traditional NN, such as long training time, slow convergence speed, ease to fall into a local minimum, and makes it have superior performance quality, and it has received good results in application.

## 2. Related Work

Prediction analysis is the process of speculating and predicting unknown future events based on known past and present events, as well as providing qualitative and quantitative descriptions of some uncertain factors and unknown events that may occur during the course of the predicted events. In general, an enterprise's sales forecast is the process of using past sales volume, sales amount, profit, and other indicators in the system to calculate future demand indicators using an algorithm mining model. Currently, a large number of academics are researching enterprise sales forecasting. The related literature of sales forecasting theory at home and abroad is discussed in this chapter, as are the benefits and drawbacks of various methods, as well as the research methods and ideas of this paper.

Arinaminpathy et al. proposed a method to build a data warehouse based on historical drug sales data and established a drug sales forecast model [10]. The improved NN by Yucesan et al. made self-calibration with reference to the prediction of synchronous time series and used a genetic algorithm to achieve self-optimization through calibration, simplifying the network structure [11]. Lian et al. took the preliminary prediction results as the input of the improved BPNN, then trained and predicted on this basis, and constructed a combined prediction model based on the improved BPNN [12]. Aiming at the characteristics of a company's drug sales, inventory, and the main problems in the sales process, Duan et al. proposed a hierarchical clustering model for multivariety drug sales forecast to predict the company's drug sales [13]. Russo et al. proposed a combinatorial optimization grey ANN model for seasonal load forecasting. The trend is predicted with a grey model; the seasonality is predicted with NN; finally, the optimal combination is carried out [14]. Aiming at some of the problems of BPNN, Curtis et al. added a momentum term to improve learning efficiency and avoid local minima [15]. Shibata et al. proposed a model for time series forecasting based on the discounted least-squares idea, and the discount is reflected in the constructed error function [16]. The discounted least squares error function biases the NN learning towards more recent observations and contemporaneous observations, while also taking into account long-term trends in the time series. Urquhart proposed an improved extreme learning machine algorithm combining batch processing and successive iterations and applied it in medium and long-term forecasting [17]. This method can improve the recognition performance of ANN in extension ability by continuously updating the training sample set. Ali and Pinar proposed two prediction models based on information fusion: one is a fusion model based on the weighted average of multiple models; the other is a fusion model that uses the advantages of various algorithms to complement each other [18]. Hassani et al. proposed an improved sales forecasting algorithm based on NN [19]. Stormi et al. studied the research on sales forecasting of the model combining NN, genetic algorithm, and PSO (particle swarm optimization) algorithm in intelligent algorithms [20].

Based on the previous research results on drug sales forecast, this paper puts forward a new research perspective and method. Because NN can approximate a nonlinear function with arbitrary precision, it can well establish the functional mapping relationship between sales volume and its various influencing factors. In this paper, using PSONN, a prediction model is constructed. A drug sales forecasting algorithm based on chaotic PSONN is proposed to improve the PSO algorithm. Inert particles tend to appear in the later stage of optimization and converge to the local optimal point prematurely. The simulation results show that the prediction effect of this model is better than that of the traditional NN model. The model and research method established in this paper are practical and effective, which not only simplifies the network structure and improves the convergence speed but also improves the prediction accuracy.

## 3. Methodology

### 3.1. NN Algorithm and Sales Forecast

ANN processor has the natural characteristics of storing and applying empirical knowledge, which is similar to that of the human brain. ANN is mainly trained by two learning algorithms, namely, the guided learning algorithm and nonguided learning algorithm. Among them, the representative BPNN is a multilayer feedforward network with hidden layers, which is a kind of error back propagation [21]. BPNN is a very effective intelligent forecasting method. Generally, BPNN is a multilayer feedforward NN, and its transfer function usually adopts the Sigmoid function. This algorithm consists of the forward transmission of information and the backward propagation of error. Its basic principle is to constantly revise the weights and thresholds of nodes at all levels in the network until the output of the network reaches the target output value, and it has good generalization ability. BPNN uses the error between the output response of the network to the learning signal and the expectation as the tutor signal and adjusts the network connection strength. Through repeated adjustments, the error is minimized, thus completing the learning process.

**Figure 1:**
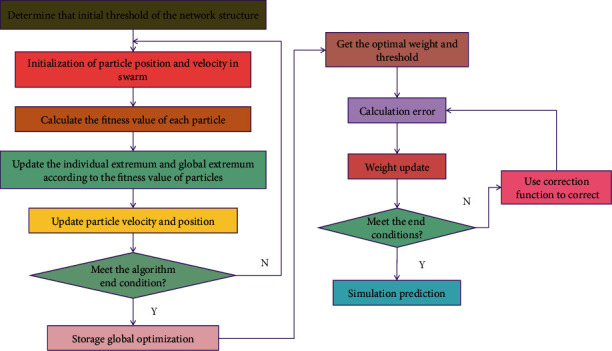
Flow chart of BPNN.

The learning process of BPNN is divided into two stages: ① input the known learning samples and calculate the output of each neuron backward from the first layer of the network through the set network structure and the weights and thresholds of the previous iteration; ② modify the weights and thresholds, calculate the influence of each weight and threshold on the total error from the last layer forward, and modify the weights and thresholds accordingly. Each neuron of NN can calculate and process independently according to the received information and then transmit the output results to other neurons for parallel processing. In the training process, as long as the input patterns are provided to NN, NN can automatically adapt to the connection weights, so that similar features can group the input patterns together. The sales forecast is an important part of the enterprise decision support system. The sales forecast is to take the purchase and sale of commodities in the market as the main object, to foresee and speculate on the changes in various purchase and sale activities, prices, and competitive conditions of commodities, and to take advantage of the situation and results. For the current sales industry, it is very important to forecast the sales of commodities [22], but the difficult problem is how to forecast the sales quickly and efficiently, but it is not easy. Traditional sales forecasting methods are mainly divided into qualitative sales forecasting methods and quantitative sales forecasting methods. In recent years, the quantitative sales forecasting method is widely used because of its objectivity and high interpretability [23]. In fact, the sales forecast of products can be regarded as a process of searching for “knowledge.” It is mainly a process of fitting the forecast model and outputting the forecast results of sales through visual software based on the data information of related commodity sales in the past and converting the original and superficial information into the relevant “knowledge” we want. Since sales forecast is to forecast the future data demand by using the characteristics of historical data, it is an effective method to divide the data into historical data and future data at different time points and then to forecast the future data by exploring the characteristics of historical data. The conventional method has some limitations in nonlinear prediction. Recently, ANN has attracted people's attention. At present, many scholars use ANN to build sales forecast models. However, the traditional BPNN has some shortcomings, so it needs to be improved. The process of using PSOBPNN in this paper is shown in Figure 1.

The network parameters and size are related to the properties of NN used for prediction. The number of neurons, the number of hidden layers, and the connection mode are all part of the network structure. The training process for a given structure consists of adjusting the parameters to obtain an approximate basic relation. The root mean square error is used to define the error, and the training process can be thought of as an optimization problem. PSO is a population-based optimization method in which the population is referred to as a particle swarm and the individual is referred to as a particle. Its basic concept is based on the simulation of seabird predation behavior, such as that of seagulls. PSO outperforms traditional random methods in terms of simplicity, ease of implementation, global optimization ability, and computing speed [24]. The position of the particle in the basic PSO algorithm represents the potential solution of the optimized problem in the search space. The optimized function determines the fitness value of each particle, and each particle has a speed that determines its flying direction and distance. Particles search the solution space by following the current best particle. Each particle state is a possible solution to the optimization problem and a fitness value determined by the objective function to be optimized in the algorithm's search space. PSO has a number of advantages, including ease of implementation, a small number of parameters to adjust, quick convergence, and low computational requirements. It has been widely used and has been proven to be an effective method for solving many global optimization problems.

### 3.2. Construction of Drug Sales Forecast Model of PSONN

**Figure 2:**
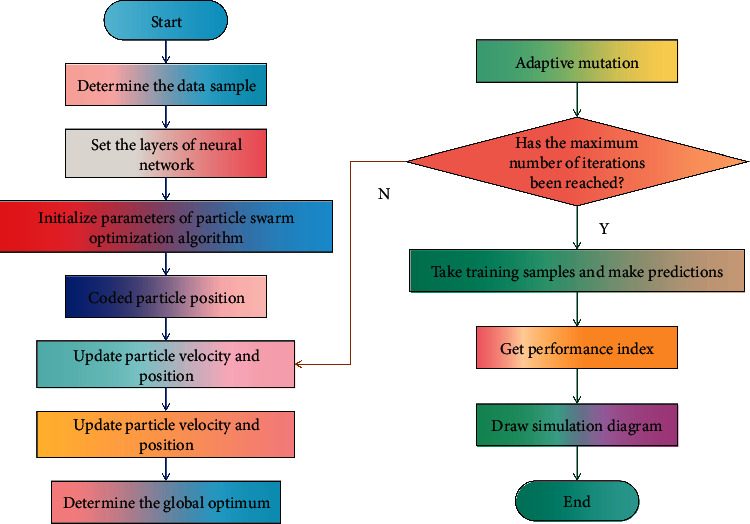
Flow chart of drug sales forecast based on PSONN.

Sales forecasting is a highly complex nonlinear system, and the modeling of sales forecasting needs the guidance of many theories, including mathematical statistics theory, artificial intelligence theory, economic theory, and nonlinear dynamics theory. In this section, PSONN is used to build a drug sales forecasting model. In this method, the weights and thresholds of BPNN are trained by PSO, the hidden rules can be automatically extracted from massive data by NN, and the advantages of global search and fast convergence of PSO are integrated to predict the economic indicators. The process of drug sales forecast based on PSONN is shown in Figure 2.

All of the weights of connections between neurons are encoded as real vectors, which are used to represent individuals in the population. The original steps of the algorithm are used to generate groups of these vectors at random and iterate them. The mean square deviation of all NN-generated samples is calculated after the newly generated individual vectors in the iteration are reduced to NN weights. The training process is stopped if it is less than the system-specified error; otherwise, it is continued until the maximum number of iterations is reached. The error information is returned according to the original route during the backward propagation process, and the weights and thresholds of NN are corrected according to the learning rules. The forward propagation process is then performed, and the two processes are repeated until the actual training output of all training samples falls within a certain error range when compared to the expected output.

The emphasis of BPNN is network error and weight adjustment. When the network output is not equal to the expected output, there is an output error *E*, which is defined as follows:

(1) $$\begin{array}{c} E=\frac{1}{2}{\left(d-o\right)}^{2}=\frac{1}{2}\sum_{k=1}^{l}{\left(d_{k}-o_{k}\right)}^{2}. \end{array}$$

Expand the above error definition to the hidden layer as follows:

(2) $$\begin{array}{c} E=\frac{1}{2}\sum_{k=1}^{l}{\left[d_{k}-f\left({\text{net}}_{k}\right)\right]}^{2}=\frac{1}{2}\sum_{k=1}^{l}{\left[d_{k}-f\left(\sum_{j=0}^{m}w_{jk}y_{i}\right)\right]}^{2}. \end{array}$$

Expand further to the input layer as follows:

(3) $$\begin{array}{c} E=\frac{1}{2}\sum_{k=1}^{l}{\left[d_{k}-f\left(\sum_{j=0}^{m}w_{jk}y_{i}\right)\right]}^{2}=\frac{1}{2}\sum_{k=1}^{l}\left\{d_{k}-f{\left[\sum_{j=0}^{m}w_{jk}f\left(\sum_{i=0}^{n}v_{ij}x_{i}\right)\right]}^{2}\right\}. \end{array}$$

It can be seen from the above formula that the network input error is a function of the weights *w*_*jk*_ and *v*_*ij*_ of each layer, so the weights can change the error *E*.

In this paper, we first consider setting up only one hidden layer. When the number of hidden nodes in a hidden layer is too large to improve the network performance, we only consider adding another hidden layer. We determine the predetermined error requirements, the maximum allowed iteration steps, the search range, the maximum speed, and the number of particles according to the network scale. In order to avoid the phenomenon of weight oscillation and slow convergence in the learning process, it is necessary to consider the influence of the previous weight change on the current weight change and add the momentum factor.

Given the original data sequence with variable *x*^(0)^,

(4) $$\begin{array}{c} x^{\left(0\right)}=\left[x^{\left(0\right)}\left(1\right),x^{\left(0\right)}\left(2\right),\ldots ,x^{\left(0\right)}\left(n\right)\right]. \end{array}$$

Generate a first-order cumulative generation sequence as follows:

(5) $$\begin{array}{c} x^{\left(1\right)}=\left[x^{\left(1\right)}\left(1\right),x^{\left(1\right)}\left(2\right),\ldots ,x^{\left(1\right)}\left(n\right)\right]. \end{array}$$

Among them,

(6) $$\begin{array}{c} x^{\left(1\right)}\left(k\right)=\sum_{i=1}^{k}x^{\left(0\right)}\left(i\right). \end{array}$$

Since the sequence *x*^(1)^(*k*) has an exponential growth law and the solution of the first-order differential equation is exactly the solution of the exponential growth form, it can be considered that the *x*^(1)^ sequence satisfies

(7) $$\begin{array}{c} \frac{\text{d}x^{\left(1\right)}}{\text{d}t}+ax^{\left(1\right)}=u. \end{array}$$

In the formula, *α* and *u* are parameters and *u* is called the control term. The solution to this differential equation is

(8) $$\begin{array}{c} x^{\left(1\right)}\left(k+1\right)=\left[x^{\left(0\right)}\left(1\right)-\frac{\overset{⌢}{u}}{\overset{⌢}{\alpha }}\right]e^{-\overset{⌢}{\alpha }k}+\frac{\overset{⌢}{u}}{\overset{⌢}{\alpha }}, \end{array}$$

where $\overset{⌢}{\alpha }$ and $\overset{⌢}{u}$ are approximate solutions of equation (7).

When learning BPNN, the initial value of its weight is very important. If the initial value is too large or too small, it will affect the learning speed, which is a complicated process of parameter system optimization. In this paper, the weights are coded by real numbers, the initial weights are set to random numbers, each particle represents the initial distribution of weights of NN, and the maximum speed limit of particles is set. We set the number of neurons in the input layer, hidden layer, and output layer of the NN. The connection weights *w*_*ij*_ and *w*_*j*_ to be optimized, the scaling factor *a*_*j*_, and the translation factor *b*_*j*_ are taken as the position vector of each particle, which is

(9) $$\begin{array}{c} \text{present}\left(m\right)=\left[w_{ij},w_{j},a_{j},b_{j}\right]. \end{array}$$

Among them, *m* is the number of particle swarms. We calculate the initial fitness value as follows:

(10) $$\begin{array}{c} F=\sum_{i=1}^{n}\left|y_{i}-h_{i}\right|. \end{array}$$

We update the record that each particle has searched for the best position so far and the global search for the best position. The fitness function is the absolute sum of the prediction errors of the NN. *n* is the total number of network output nodes, *y*_*i*_ is the predicted output of the *i* th node, and DD is the expected output of the *h*_*i*_ th node. In this section, PSO is introduced, and by using its good algorithm convergence, the problem can converge to the global optimal solution or suboptimal solution with high probability, which solves the local convergence problem of BPNN itself. At the same time, the weights and thresholds of BPNN can be optimized, and the accuracy of BPNN in economic index prediction can be improved.

## 4. Result Analysis and Discussion

This section will introduce the data testing scheme and design process of the PSONN prediction model in detail. It will also test the model through actual data sets and compare it with other commonly used and similar models. The experimental results are discussed and analyzed. In this paper, Matlab software is used for simulation and prediction. Firstly, we design the structure and parameters of the network. The parameters to be determined in this paper are the number of nodes in the input layer and output layer of the network, the number of hidden layers, the number of nodes in hidden layers, the initial weights, etc. We set the number of nodes in the middle layer to 25, the hidden layer of the model to 30, and the weight learning rate to 0.2. The number of neurons in the input layer is 10, and the number of neurons in the output layer is 1. When the number of hidden neurons is 16, the model has the best prediction effect. We initialize the parameters of the PSO algorithm, with iteration times of 200 and a population number of 30. We take the sales data of a pharmaceutical company in 2019 as the original test data. The data are classified into training sample data and test data. Three terms of “sales time,” “commodity number,” and “sales quantity” are extracted from the test data, and the original sales details data are preliminarily screened and then summarized. The results are shown in Table 1.

**Figure 3:**
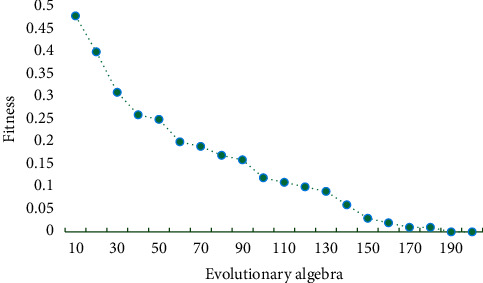
PSO process of adjusting inertia weight.

In this paper, the original data are normalized, that is, the input and output data are uniformly limited to [0, 1] or [−1,1] by a certain linear transformation. The normalization process makes the drug sequences with different units and dimensions mapped into the [0,1] interval, which eliminates the influence of different dimensions on the input data and speeds up the training speed of NN and its related algorithms. The PSONN model is tested by using the preprocessed drug sales series. A simple mutation operator is added to reinitialize the particles with a certain probability, and the optimization is carried out by cyclic iteration until the maximum number of iterations is reached, and the optimal individual fitness value is recorded. The variation process of the optimal individual fitness value in the optimization process based on the adjusted inertia weight PSONN is shown in Figure 3.

**Figure 4:**
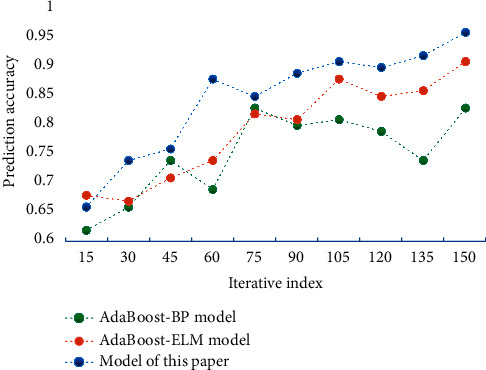
Comparison of prediction accuracy of different models.

**Figure 5:**
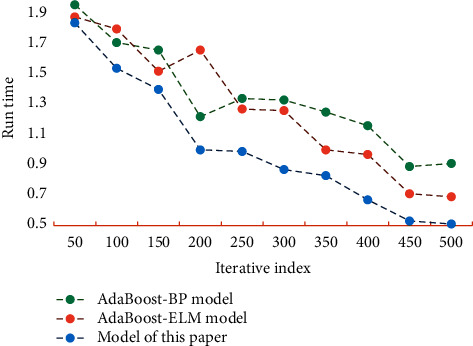
Comparison of running time of different models.

From Figure 3, it can be seen that the PSO algorithm has a strong optimization ability, and the optimization of the algorithm is faster at the initial stage of iteration. Through the construction and testing of the model proposed in this paper, the AdaBoost-BP model and AdaBoost-ELM model, the prediction accuracy of each drug, the average accuracy of all drugs, and the total running time of the three models are obtained.  Figure 4 shows the comparison of prediction accuracy of different models.  Figure 5 shows the running time comparison of different models.

As can be seen from Figure 4, the sales forecasting accuracy of this model is higher than that of the other two models, and it is at a high level. The prediction accuracy of this model can reach 96.14%, which is about 12.78% higher than that of the traditional BP model. From the analysis of Figure 5, it can be seen that the running time of this model is short and its efficiency is high. Therefore, the sales forecasting method based on PSONN proposed in this paper has certain superior performance and has certain reliability and practicability.

**Figure 6:**
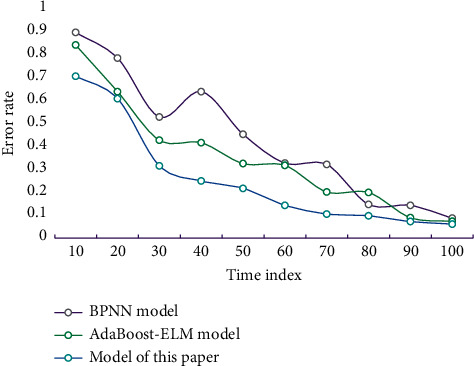
Error comparison of different models.

The connection weights and thresholds are fixed in the prediction knowledge base. At this time, the whole NN is a time series prediction model, and the performance of the model is tested by test samples. The prediction error defined in this paper is the difference between the predicted value and the actual value.  Figure 6 shows the error comparison of different models.

It can be seen that, on the whole, the error rate of this model is at a low level, indicating that the accuracy of this method is high.  Table 2 shows the specific situation of the corresponding predicted performance indicators.

**Figure 7:**
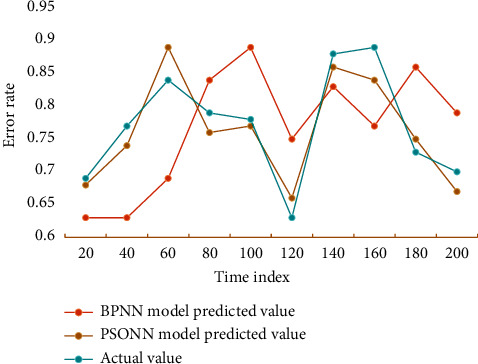
Simulation diagram of the drug sales forecast.

It can be seen from the data in the table that the overall trend between the predicted value and the actual value is consistent, which indicates that it is feasible to forecast the drug sales based on PSONN. The simulation diagram of drug sales forecast based on PSONN and traditional BPNN is shown in Figure 7.

It can be seen from the prediction chart that the predicted value based on PSONN is closer to the actual value, and its prediction effect is better than that of traditional BPNN. It shows that the improved algorithm is better than the traditional prediction model. A large number of experimental results in this chapter show that the NN sales forecasting model optimized by PSO can achieve 96.14% forecasting accuracy, which is about 12.78% higher than the traditional BP model. Using PSO instead of the traditional training method of gradient descent of NN improves the dilemma that NN easily falls into the minimum value, improves the convergence speed, and makes the model have a certain superior performance.

## 5. Conclusions

The sales link, which connects suppliers, sellers, and consumers, is an important link in enterprise management. The most important part of the sales process is sales forecasting, and most commodity circulation industries' supply chains have inventory and sales problems due to a lack of appropriate sales forecasting methods. ANN has some advantages in dealing with nonlinear problems because it can cluster and learn from a large amount of historical data and then find some rules of behavior changes. As a result, the BPNN model is chosen as the basic forecasting model for drug sales forecasting, as it is the most widely used among the NN models. PSO is used to overcome the drawbacks of traditional NN, such as long training times, slow convergence speeds, and the ease with which it falls into local minima, resulting in improved performance. Experiments show that the PSO-optimized NN sales forecasting model can achieve a forecasting accuracy of 96.14 percent, which is about 12.78 percent higher than the traditional BP model. This model addresses traditional NN's flaws, such as long training times, slow convergence speeds, and a proclivity to fall into local minima, among others, and improves performance quality, yielding positive results in practice. The research shows that the forecasting method presented in this paper has a better forecasting effect on drug sales forecasting and that using the optimized model presented in this paper to forecast drug sales is practical and feasible. It is a good starting point for creating a prediction model for other problems. Although this model's prediction accuracy and run time have clearly improved, it still has some limitations. PSONN and particle swarm improvement have randomness, which makes prediction stability poor. In addition, the model comparison does not include the testing of some algorithms related to sales forecasting in this paper. I hope that the optimization algorithm will be further investigated in future work and research and that the algorithm's stability will be improved, resulting in a more perfect and reasonable model.

## Figures and Tables

**Table 1:** Summary of original sales details data.

Sales time	Commodity number	Sales volumes
2019-01	23769	758
2019-02	13875	544
2019-03	36974	721
2019-04	25897	473
2019-05	23521	130
2019-06	13267	167
2019-07	53679	871
2019-08	89732	165
2019-09	13078	746
2019-10	75890	752
2019-11	18970	546
2019-12	57698	655

**Table 2:** Comparison of evaluation indexes of different methods.

Method	Mean square error	Mean absolute error	Average absolute percentage error	Mean square percentage error
Traditional BPNN	18.692	3.217	0.0814	0.0169
AdaBoost-ELM	13.417	2.136	0.0689	0.0132
Support vector machine	14.685	2.987	0.0897	0.0187
PSONN in this paper	12.374	2.054	0.0657	0.0124

## Data Availability

The data used to support the findings of this study are available from the corresponding author upon request.
